# Supplementary material for: Circadian regulation of endoplasmic reticulum calcium response in cultured mouse astrocytes
Source: eLife. 2024 Nov 27;13:RP96357. doi: 10.7554/eLife.96357 (PMC11602189; doi:10.7554/eLife.96357)
Supplement: Figure 2—source data 1. [file elife-96357-fig2-data1.zip › Figure 2-source data 1.pdf]

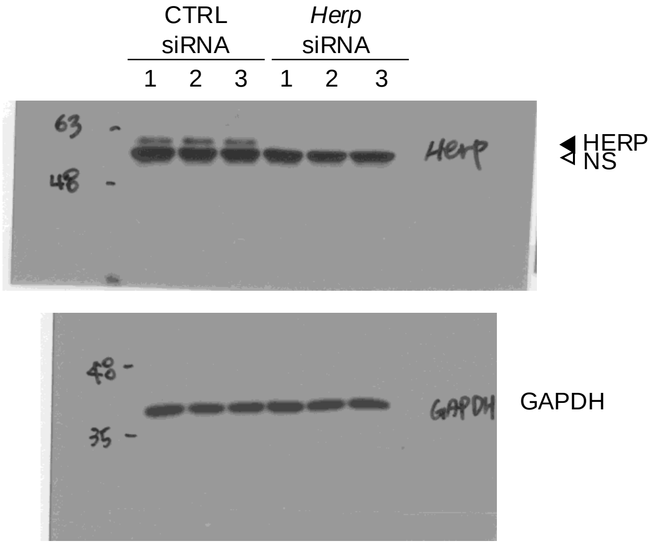

Figure2-source data 1 Original membranes corresponding to Figure 2, panel D, were used, with Gangnam-stained molecular weight markers.
