# Supplementary figures and images for: Circadian regulation of endoplasmic reticulum calcium response in cultured mouse astrocytes
Source: eLife. 2024 Nov 27;13:RP96357. doi: 10.7554/eLife.96357 (PMC11602189; doi:10.7554/eLife.96357)

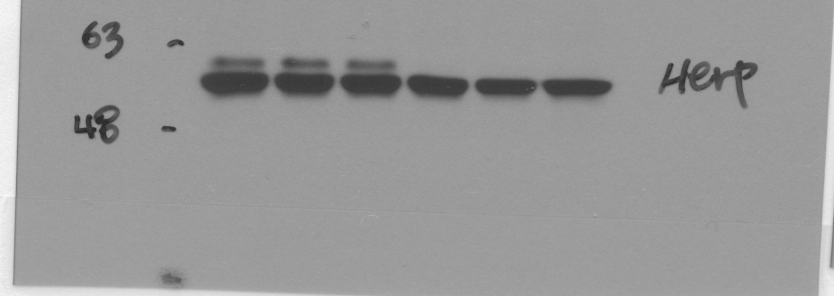

Supplement: Figure 2—source data 2. [file elife-96357-fig2-data2.zip › HERP.tif]

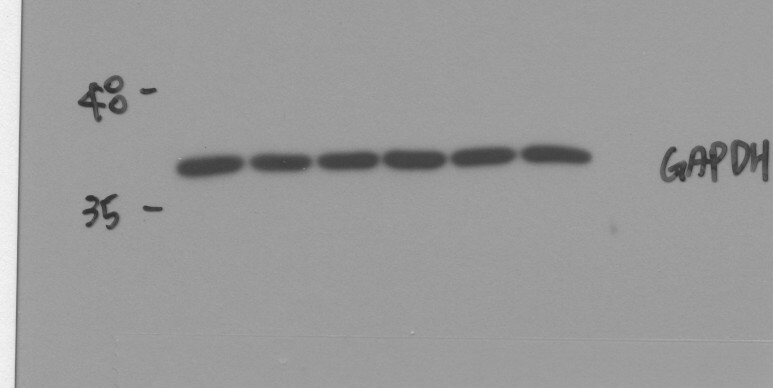

Supplement: Figure 2—source data 2. [file elife-96357-fig2-data2.zip › GAPDH.tif]

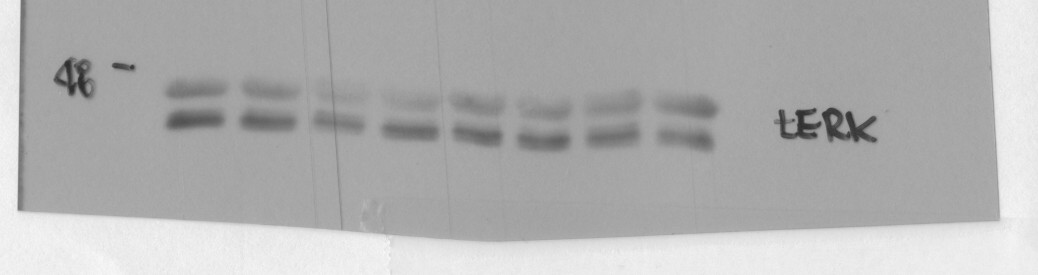

Supplement: Figure 2—source data 4. [file elife-96357-fig2-data4.zip › tERK.tif]

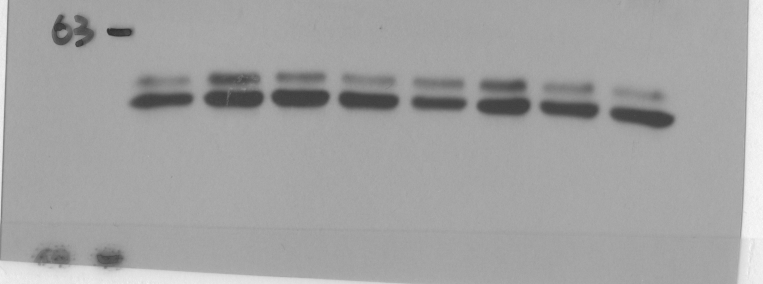

Supplement: Figure 2—source data 4. [file elife-96357-fig2-data4.zip › HERP.tif]

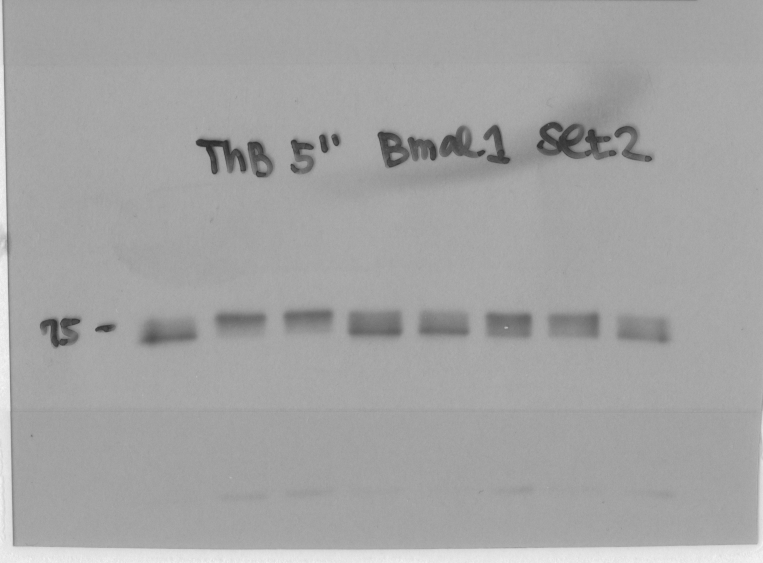

Supplement: Figure 2—source data 4. [file elife-96357-fig2-data4.zip › BMAL1.tif]

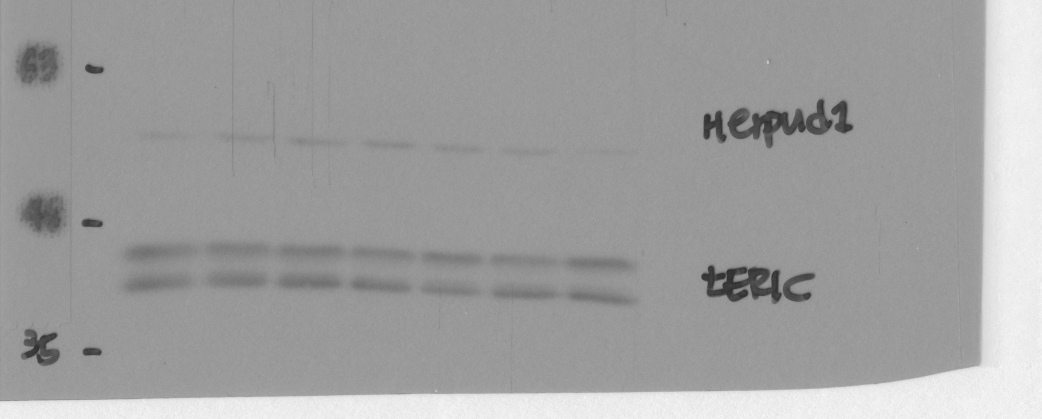

Supplement: Figure 2—source data 6. [file elife-96357-fig2-data6.zip › tERK.tif]

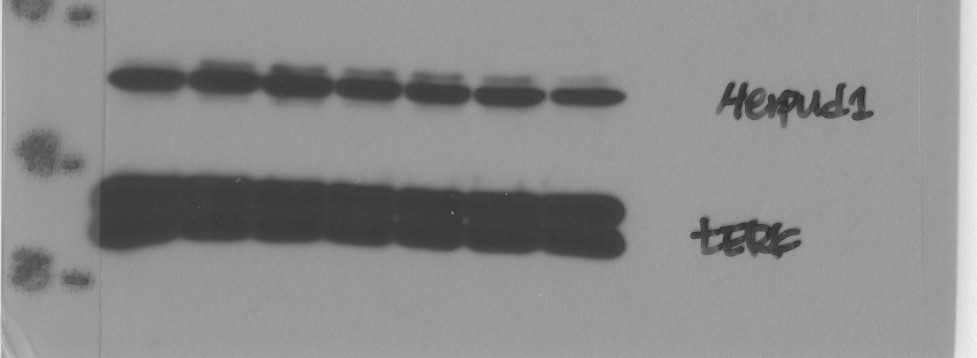

Supplement: Figure 2—source data 6. [file elife-96357-fig2-data6.zip › HERP.tif]

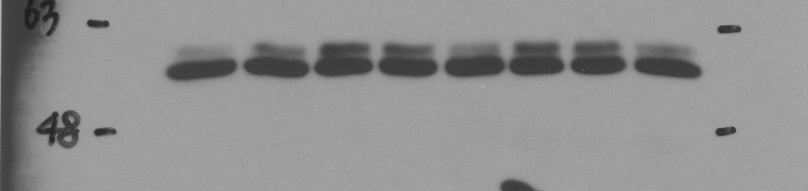

Supplement: Figure 2—figure supplement 1—source data 2. [file elife-96357-fig2-figsupp1-data2.zip › HERP.tif]

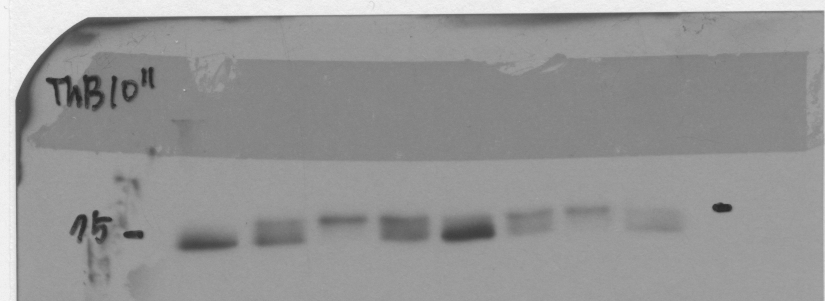

Supplement: Figure 2—figure supplement 1—source data 2. [file elife-96357-fig2-figsupp1-data2.zip › BMAL1.tif]

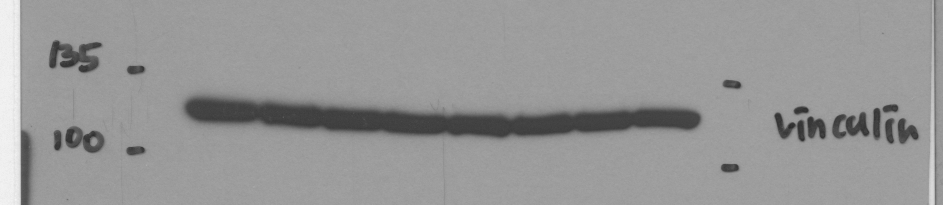

Supplement: Figure 2—figure supplement 1—source data 2. [file elife-96357-fig2-figsupp1-data2.zip › VINCULIN.tif]

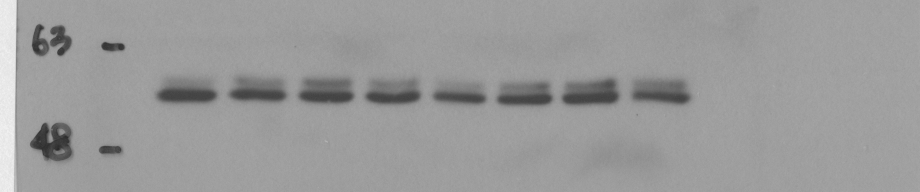

Supplement: Figure 2—figure supplement 1—source data 4. [file elife-96357-fig2-figsupp1-data4.zip › HERP.tif]

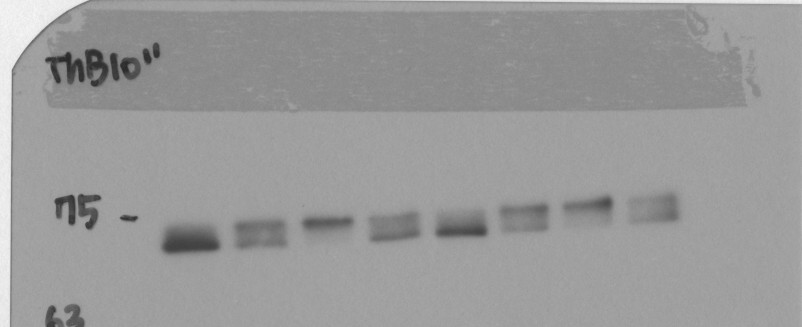

Supplement: Figure 2—figure supplement 1—source data 4. [file elife-96357-fig2-figsupp1-data4.zip › BMAL1.tif]

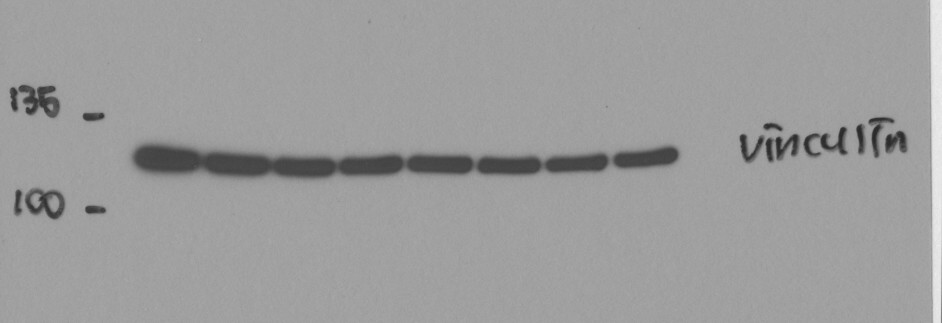

Supplement: Figure 2—figure supplement 1—source data 4. [file elife-96357-fig2-figsupp1-data4.zip › VINCULIN.tif]

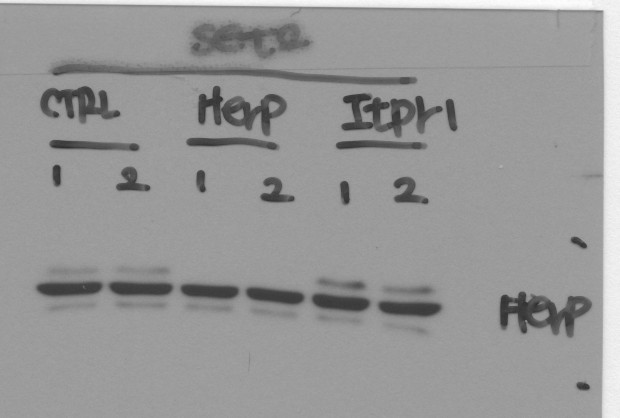

Supplement: Figure 3—source data 2. [file elife-96357-fig3-data2.zip › HERP.tif]

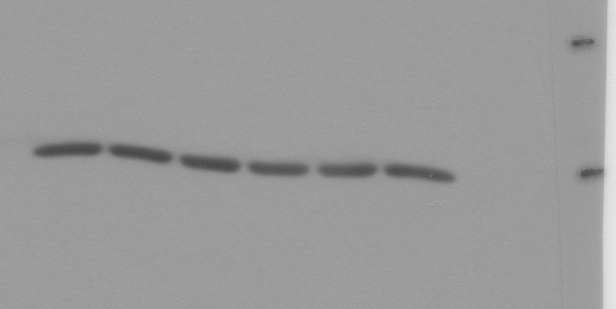

Supplement: Figure 3—source data 2. [file elife-96357-fig3-data2.zip › GAPDH.tif]

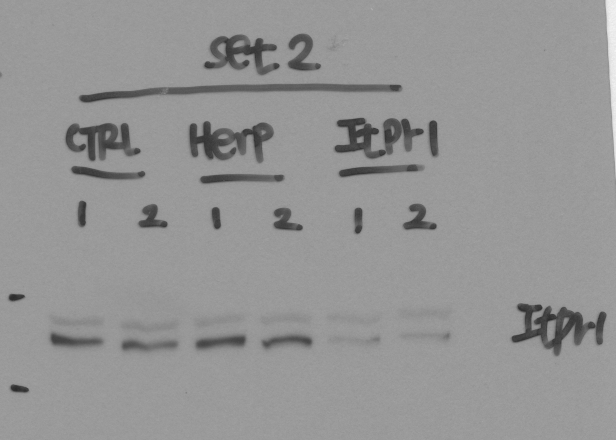

Supplement: Figure 3—source data 2. [file elife-96357-fig3-data2.zip › ITPR1.tif]

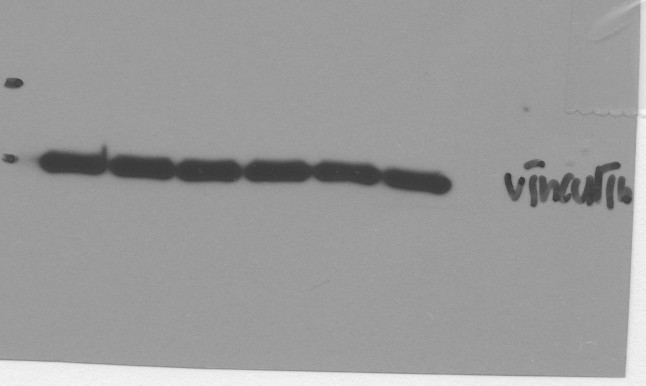

Supplement: Figure 3—source data 2. [file elife-96357-fig3-data2.zip › vinculin.tif]

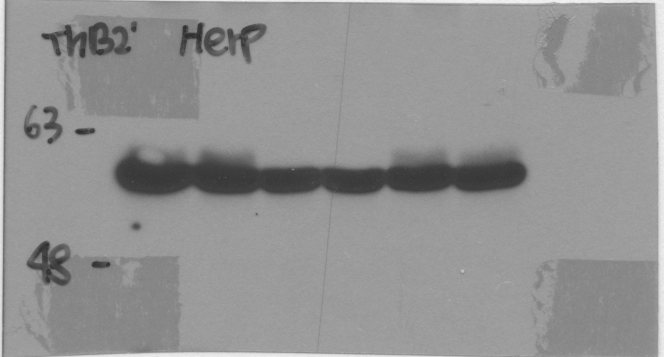

Supplement: Figure 3—source data 4. [file elife-96357-fig3-data4.zip › HERP.tif]

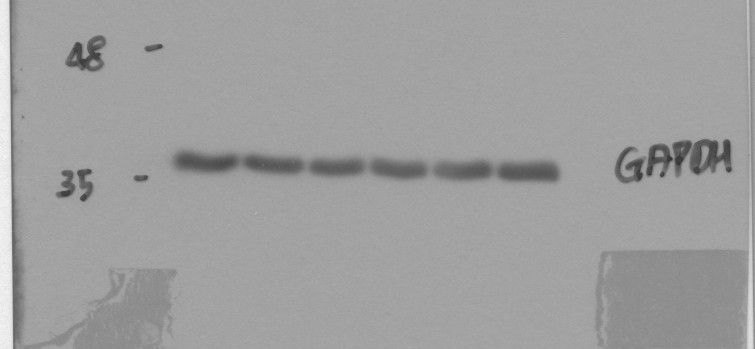

Supplement: Figure 3—source data 4. [file elife-96357-fig3-data4.zip › GAPDH.tif]

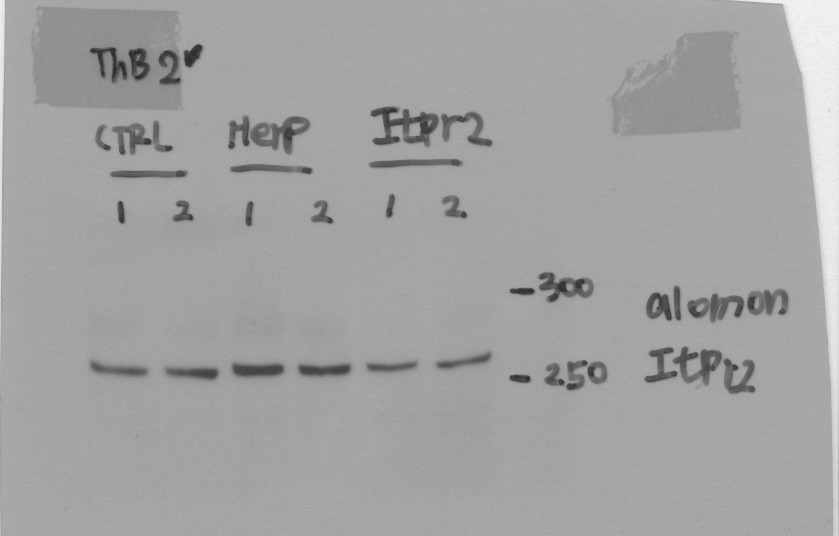

Supplement: Figure 3—source data 4. [file elife-96357-fig3-data4.zip › ITPR2.tif]

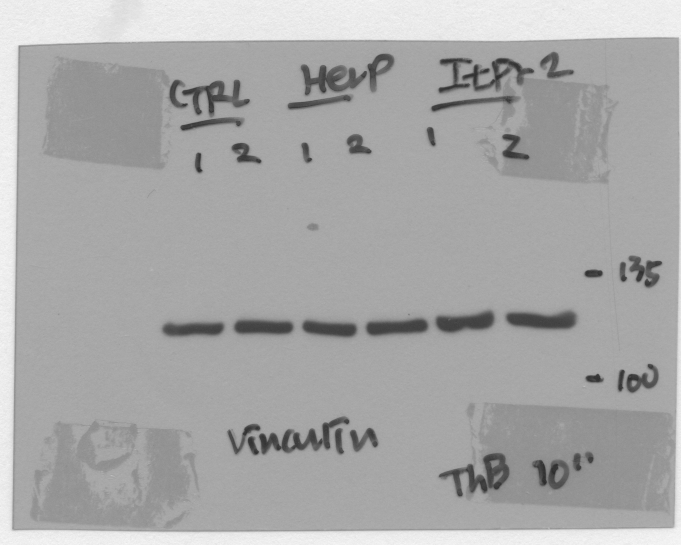

Supplement: Figure 3—source data 4. [file elife-96357-fig3-data4.zip › VINCULIN.tif]

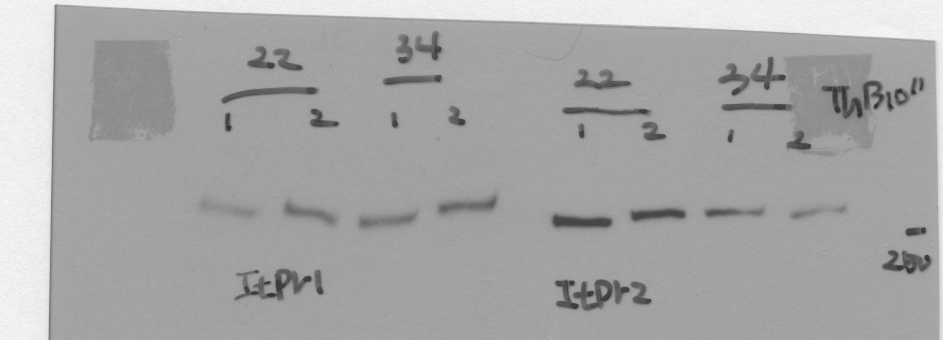

Supplement: Figure 4—source data 2. [file elife-96357-fig4-data2.zip › short expose.tif]

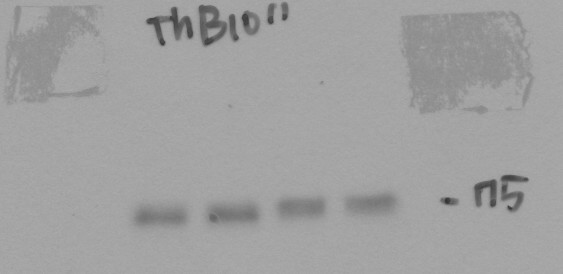

Supplement: Figure 4—source data 2. [file elife-96357-fig4-data2.zip › BMAL1.tif]

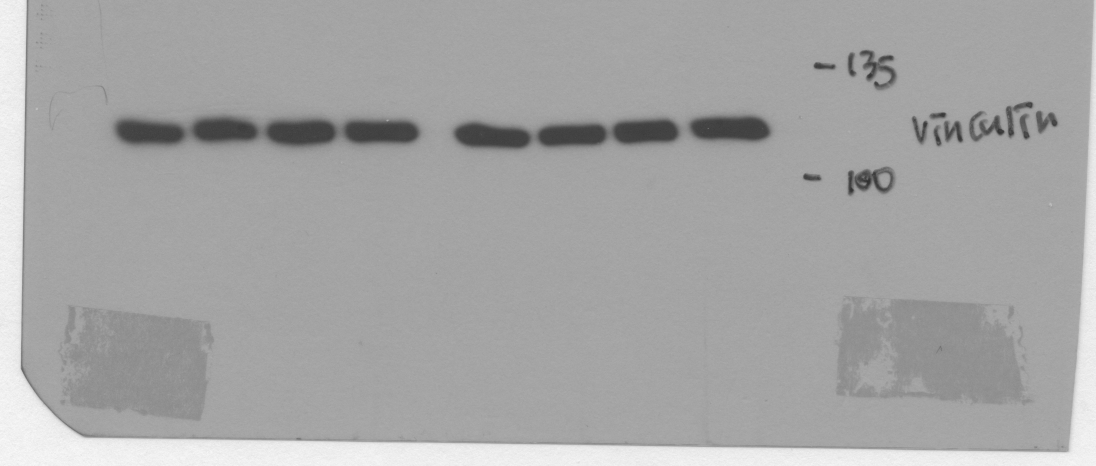

Supplement: Figure 4—source data 2. [file elife-96357-fig4-data2.zip › VINCULIN .tif]

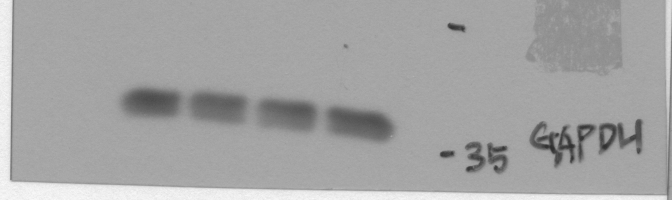

Supplement: Figure 4—source data 2. [file elife-96357-fig4-data2.zip › GAPDH.tif]

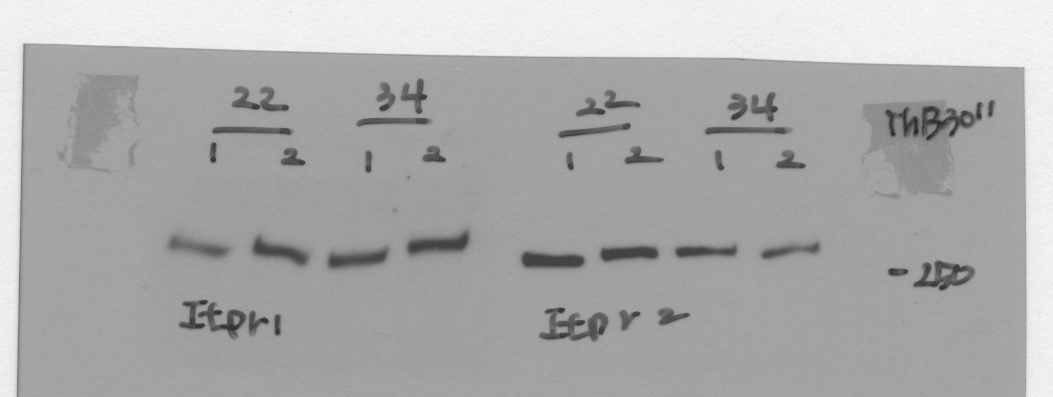

Supplement: Figure 4—source data 2. [file elife-96357-fig4-data2.zip › long expose.tif]

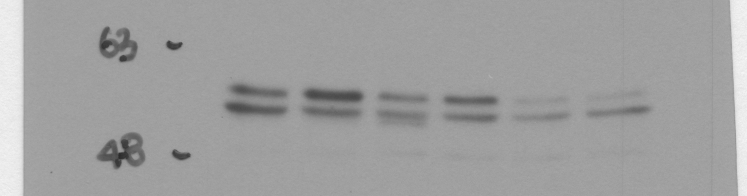

Supplement: Figure 5—source data 2. [file elife-96357-fig5-data2.zip › HERP.tif]

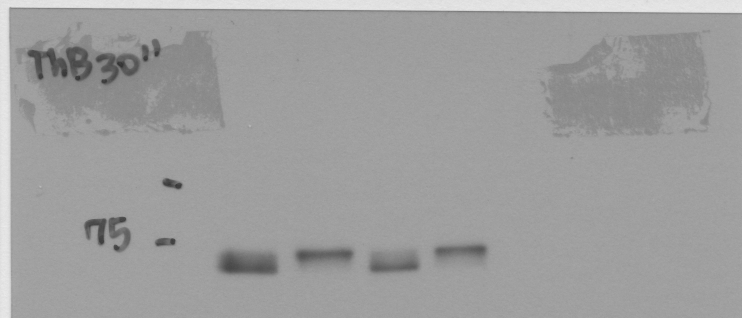

Supplement: Figure 5—source data 2. [file elife-96357-fig5-data2.zip › BMAL1.tif]

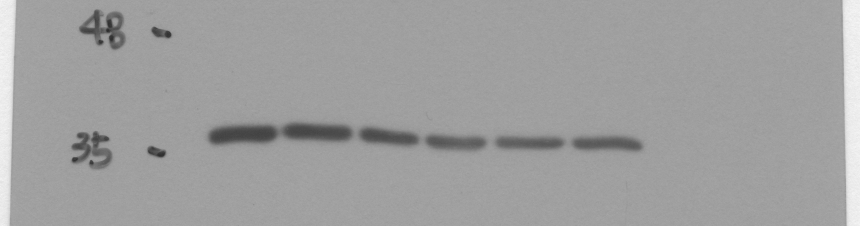

Supplement: Figure 5—source data 2. [file elife-96357-fig5-data2.zip › GAPDH.tif]

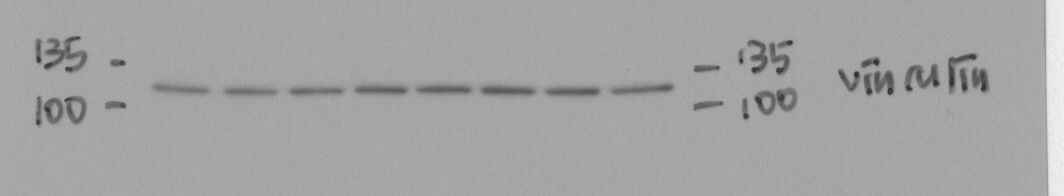

Supplement: Figure 6—source data 2. [file elife-96357-fig6-data2.zip › VINCULIN for pCX43.tif]

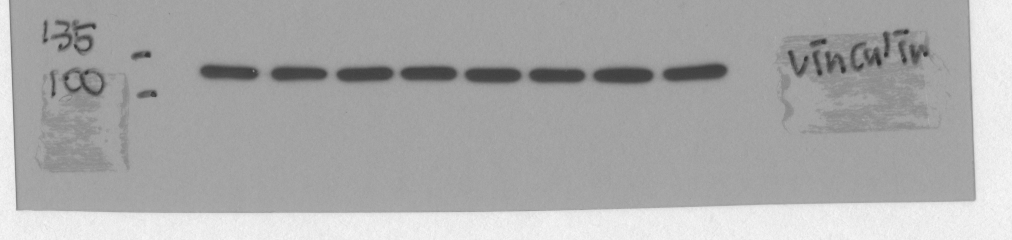

Supplement: Figure 6—source data 2. [file elife-96357-fig6-data2.zip › VINCULIN for CX43.tif]

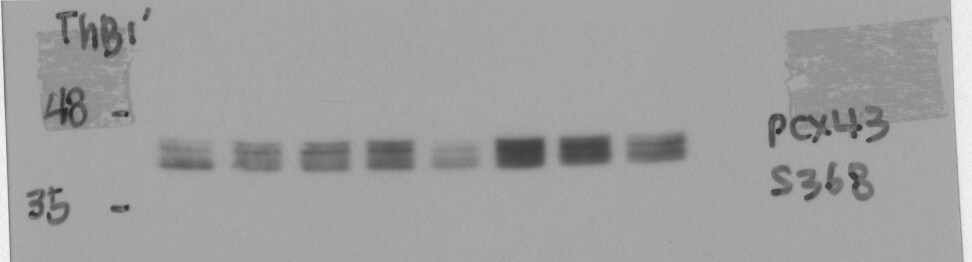

Supplement: Figure 6—source data 2. [file elife-96357-fig6-data2.zip › pCX43.tif]

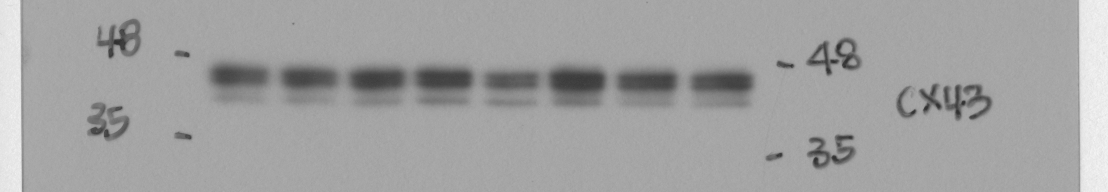

Supplement: Figure 6—source data 2. [file elife-96357-fig6-data2.zip › CX43.tif]

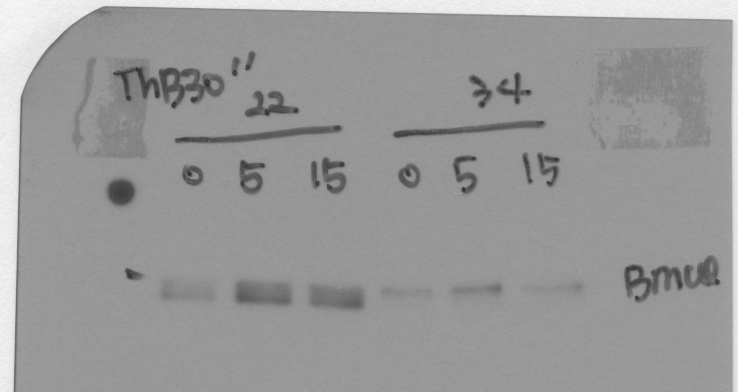

Supplement: Figure 6—source data 4. [file elife-96357-fig6-data4.zip › BMAL1.tif]

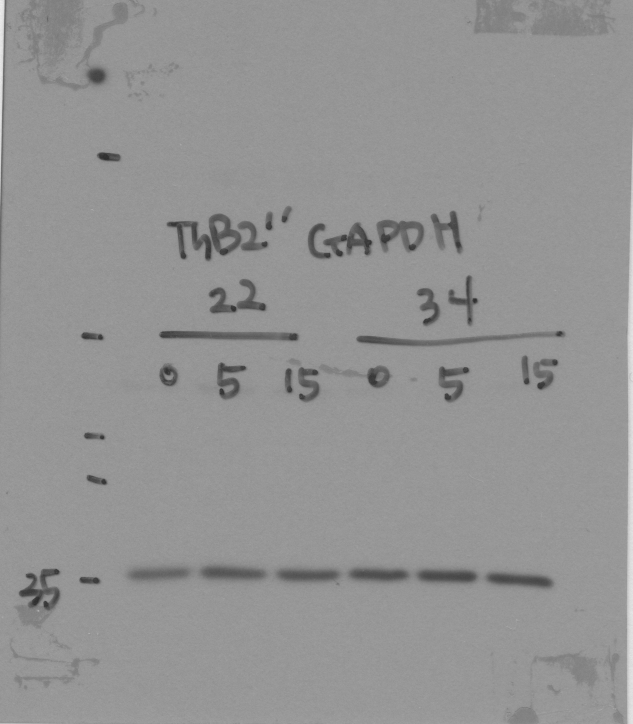

Supplement: Figure 6—source data 4. [file elife-96357-fig6-data4.zip › GAPDH.tif]

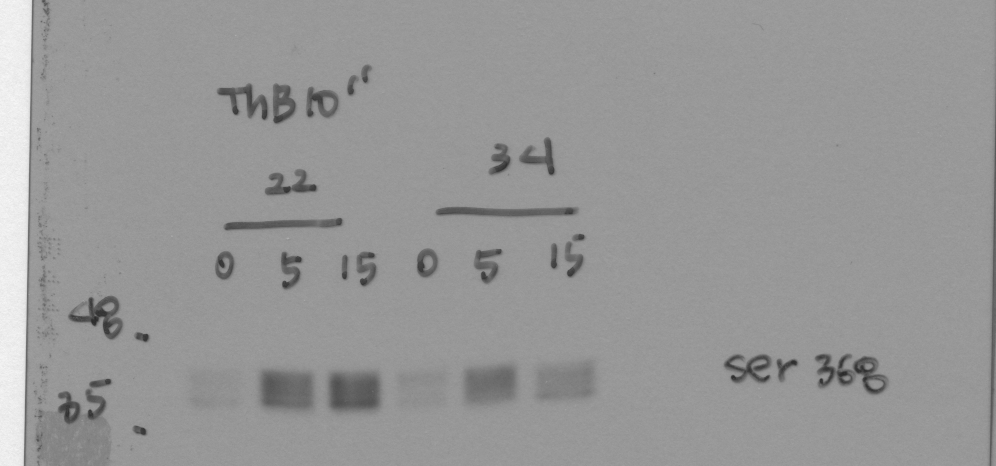

Supplement: Figure 6—source data 4. [file elife-96357-fig6-data4.zip › pCX43.tif]

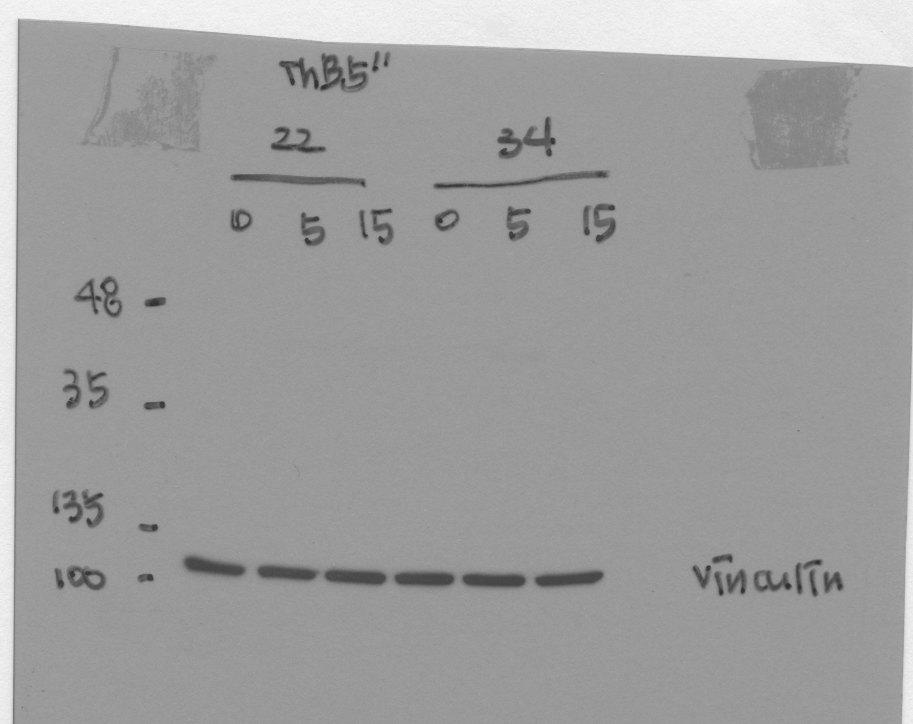

Supplement: Figure 6—source data 4. [file elife-96357-fig6-data4.zip › VINCULIN.tif]

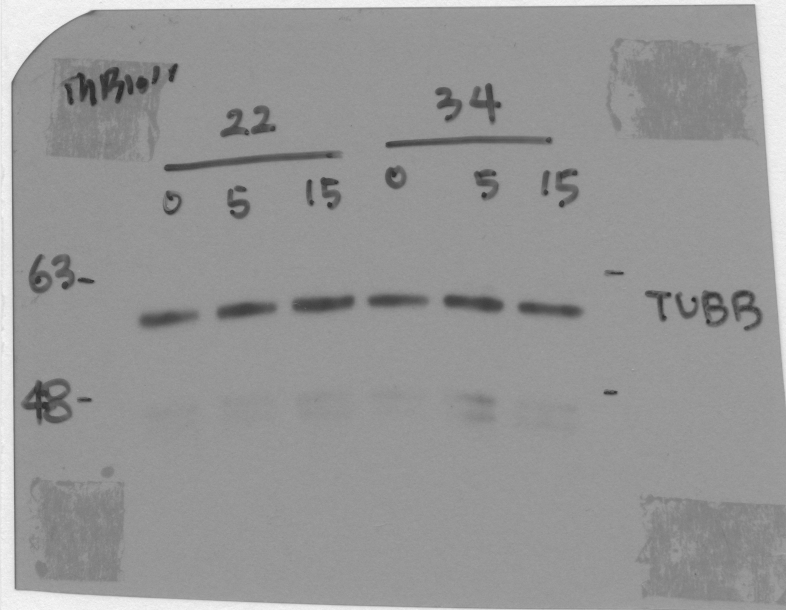

Supplement: Figure 6—source data 4. [file elife-96357-fig6-data4.zip › TUBB.tif]

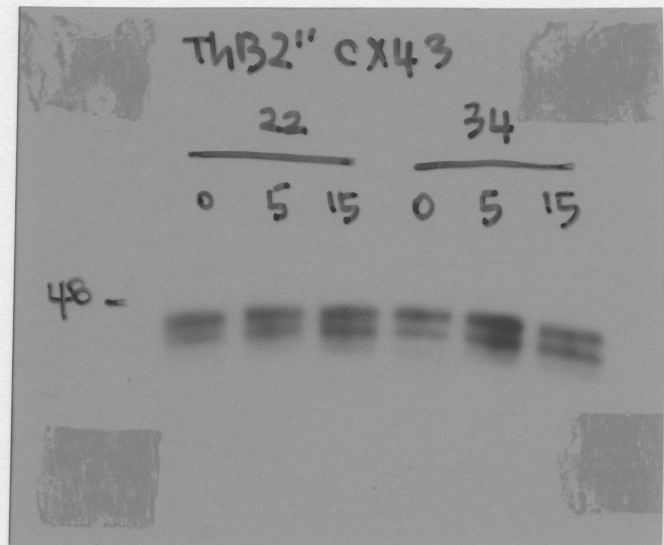

Supplement: Figure 6—source data 4. [file elife-96357-fig6-data4.zip › CX43.tif]

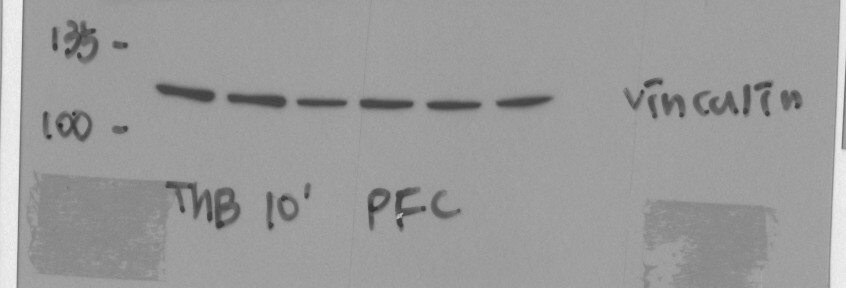

Supplement: Figure 6—source data 6. [file elife-96357-fig6-data6.zip › VINCULIN for pCX43.tif]

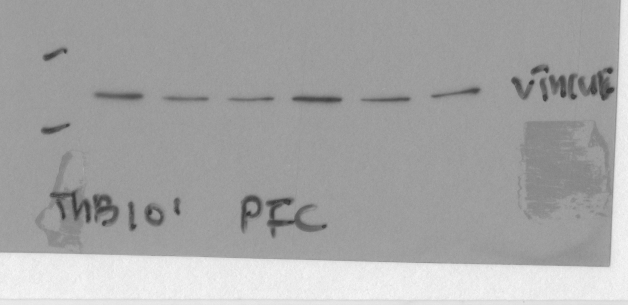

Supplement: Figure 6—source data 6. [file elife-96357-fig6-data6.zip › VINCULIN for CX43.tif]

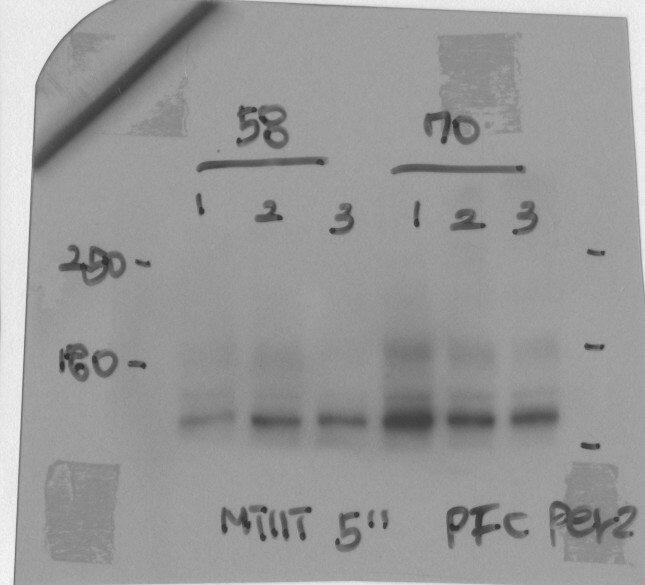

Supplement: Figure 6—source data 6. [file elife-96357-fig6-data6.zip › PER2.tif]

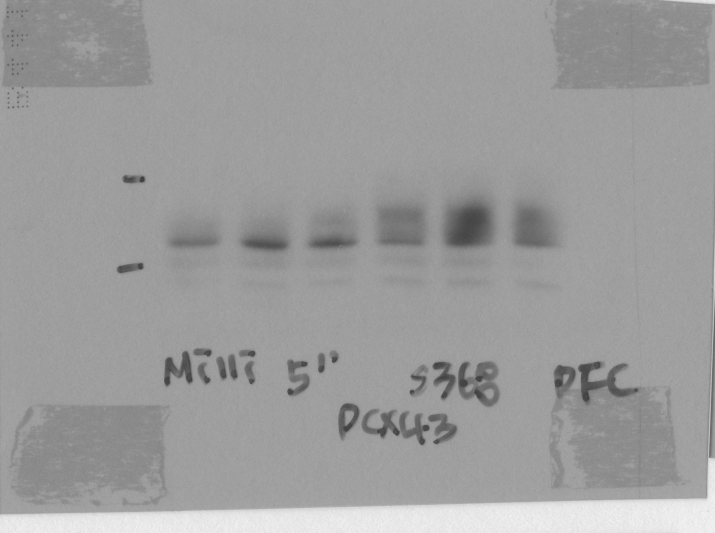

Supplement: Figure 6—source data 6. [file elife-96357-fig6-data6.zip › pCX43.tif]

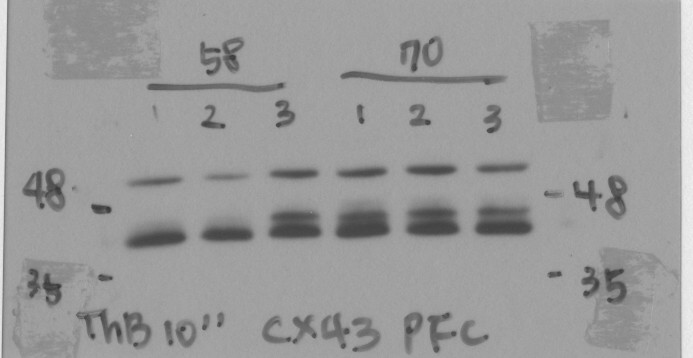

Supplement: Figure 6—source data 6. [file elife-96357-fig6-data6.zip › CX43.tif]

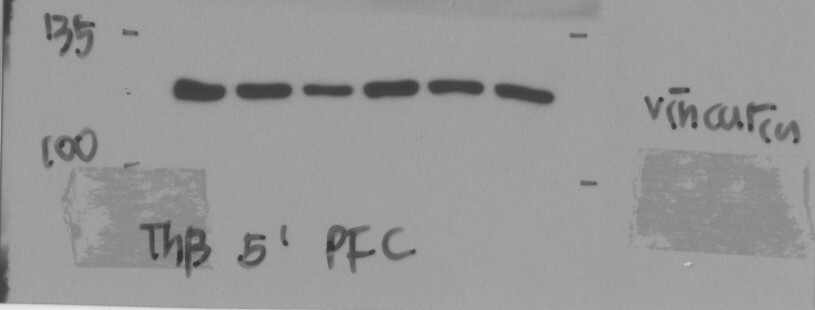

Supplement: Figure 6—source data 6. [file elife-96357-fig6-data6.zip › VINCLIN for PER2.tif]

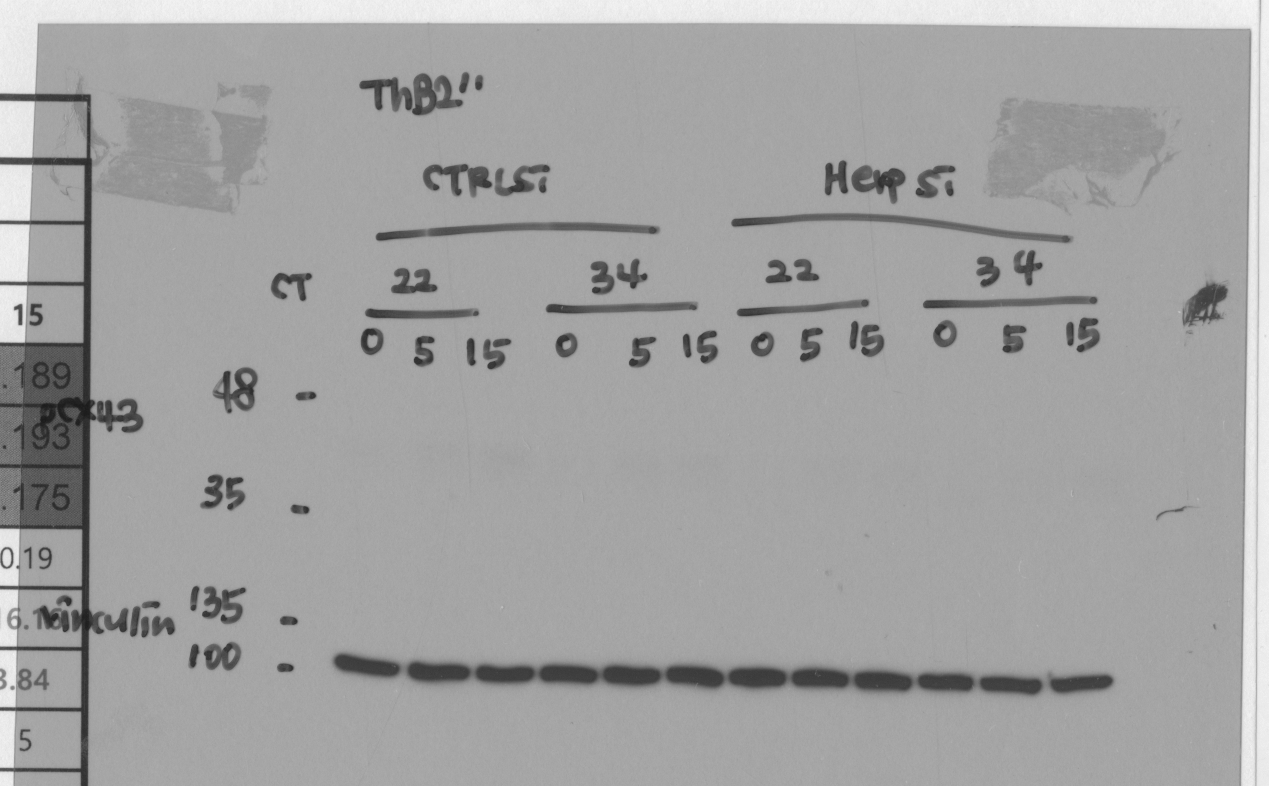

Supplement: Figure 6—source data 8. [file elife-96357-fig6-data8.zip › VINCULIN for pCX43.tif]

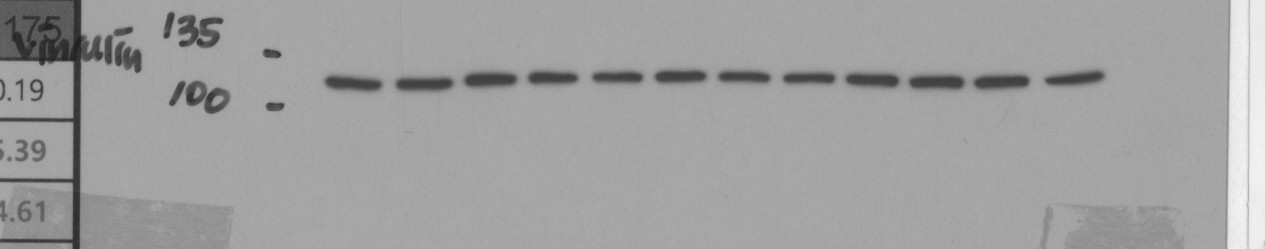

Supplement: Figure 6—source data 8. [file elife-96357-fig6-data8.zip › VINCULIN for CX43.tif]

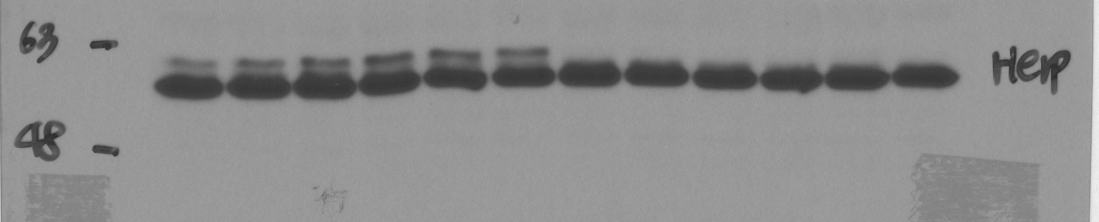

Supplement: Figure 6—source data 8. [file elife-96357-fig6-data8.zip › HERP.tif]

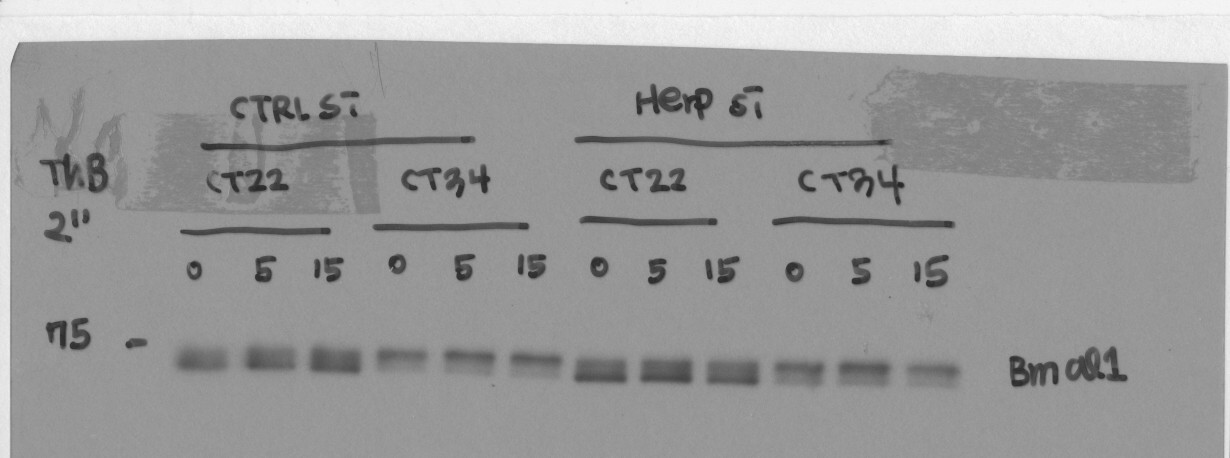

Supplement: Figure 6—source data 8. [file elife-96357-fig6-data8.zip › BMAL1.tif]

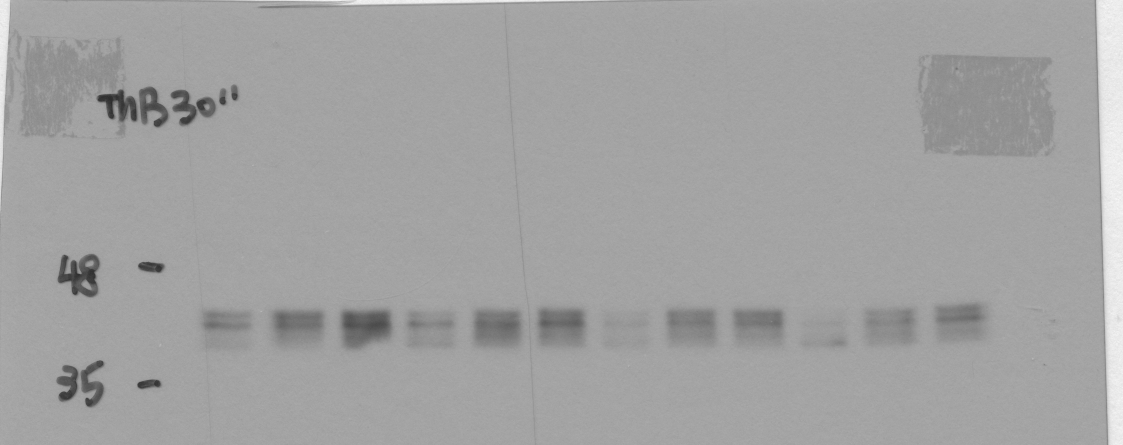

Supplement: Figure 6—source data 8. [file elife-96357-fig6-data8.zip › pCX43.tif]

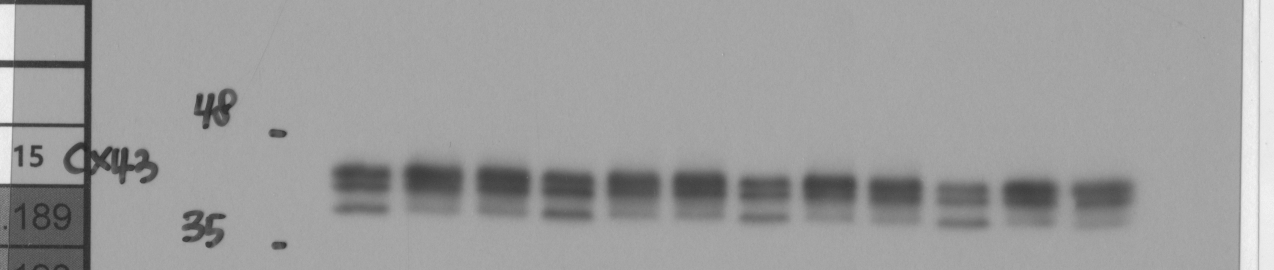

Supplement: Figure 6—source data 8. [file elife-96357-fig6-data8.zip › CX43.tif]

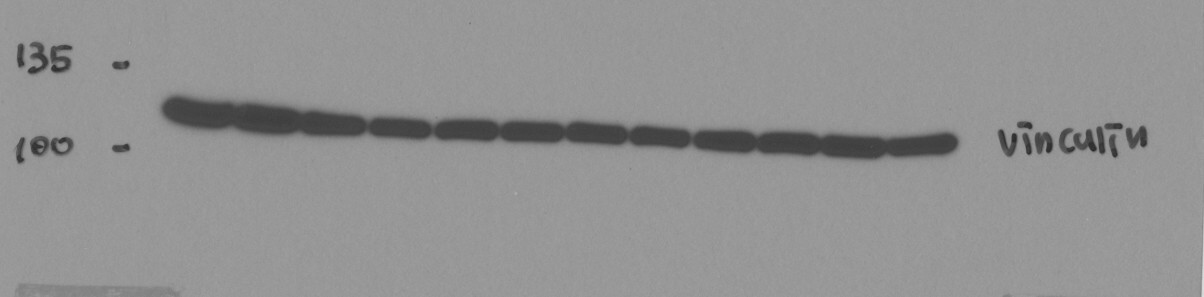

Supplement: Figure 6—source data 8. [file elife-96357-fig6-data8.zip › VINCULIN for BMAL1 and HERP.tif]
