# Supplementary material for: Circadian regulation of endoplasmic reticulum calcium response in cultured mouse astrocytes
Source: eLife. 2024 Nov 27;13:RP96357. doi: 10.7554/eLife.96357 (PMC11602189; doi:10.7554/eLife.96357)
Supplement: Figure 2—figure supplement 1—source data 3. [file elife-96357-fig2-figsupp1-data3.zip › Figure 2-Figure supplement 1-source data 3.pdf]

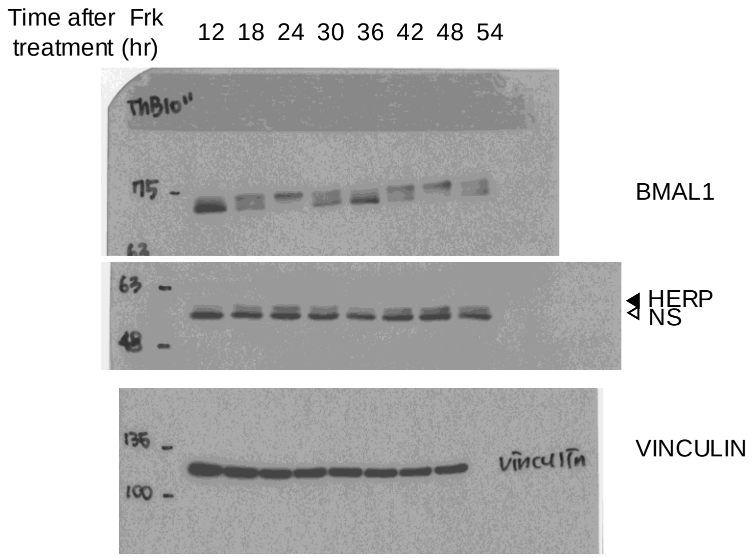

Figure 2 –figure supplement 1 -source data 3. Original membranes corresponding to Figure 2 –figure supplement 1 , panel F, were used, with Gangnam-stained molecular weight markers.
