# Supplementary material for: Circadian regulation of endoplasmic reticulum calcium response in cultured mouse astrocytes
Source: eLife. 2024 Nov 27;13:RP96357. doi: 10.7554/eLife.96357 (PMC11602189; doi:10.7554/eLife.96357)
Supplement: Figure 3—source data 1. [file elife-96357-fig3-data1.zip › Figure 3-source data 1.pdf]

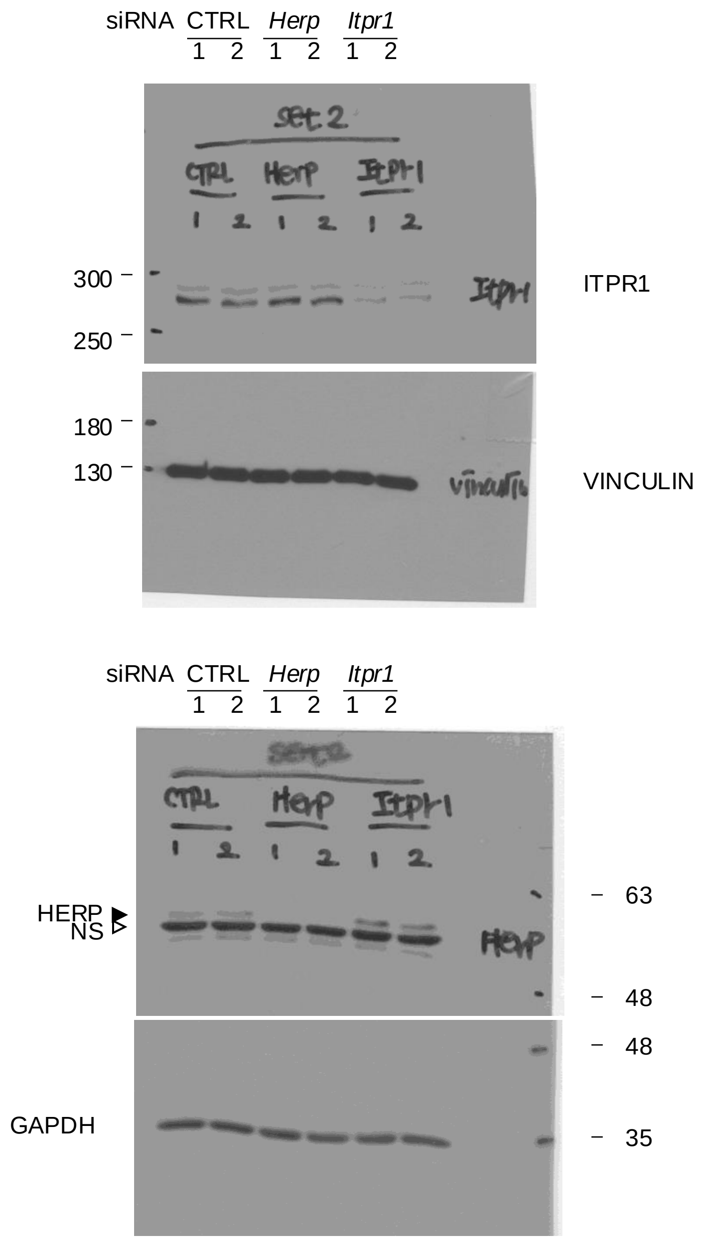

Figure3-source data 1 Original membranes corresponding to Figure 3, panel J. HERP and GAPDH were used with GangNam-STAIN molecular weight markers (iNtRON, Korea, #24052), while ITPR1 and VINCULIN were used with the Spectra™ Multicolor High Range Protein Ladder (Invitrogen, USA, #26625).
