# Supplementary material for: Circadian regulation of endoplasmic reticulum calcium response in cultured mouse astrocytes
Source: eLife. 2024 Nov 27;13:RP96357. doi: 10.7554/eLife.96357 (PMC11602189; doi:10.7554/eLife.96357)
Supplement: Figure 4—source data 1. [file elife-96357-fig4-data1.zip › Figure 4-source data 1.pdf]

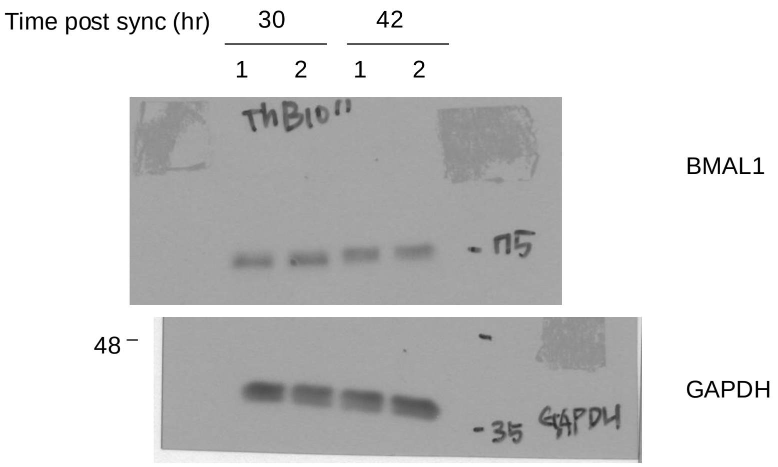

For ease of internal interpretation, we label "Time post sync (hr) 8 hr" as CT0. Therefore, the values 22 and 34 written on the scan file correspond to 30 and 42, respectively, in terms of "Time post sync (hr)."

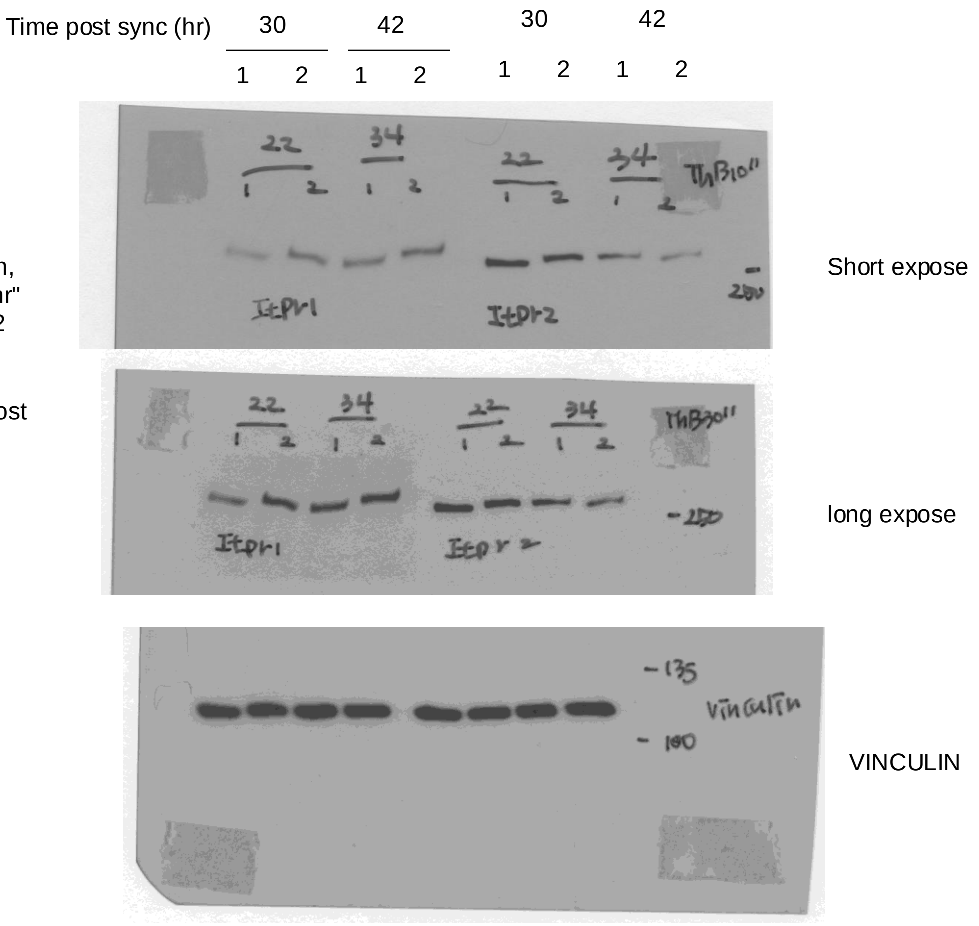

Figure 4-source data 1 Original membranes corresponding to Figure 4, panel N, were used, with Gangnam-stained molecular weight markers. The short exposure shows the raw image of ITPR1 in Figure 4, panel N, while the long exposure shows ITPR2
