# Supplementary material for: Circadian regulation of endoplasmic reticulum calcium response in cultured mouse astrocytes
Source: eLife. 2024 Nov 27;13:RP96357. doi: 10.7554/eLife.96357 (PMC11602189; doi:10.7554/eLife.96357)
Supplement: Figure 6—source data 3. [file elife-96357-fig6-data3.zip › Figure 6-source data 3.pdf]

Time post sync (hr) 30 42
ATP (min) 0 5 15 0 5 15

75 –
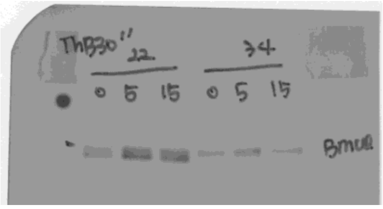
BMAL1

63 –
48 –
35 –
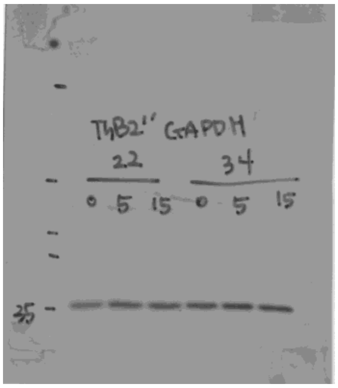
GAPDH

Time post sync (hr) 30 42
ATP (min) 0 5 15 0 5 15

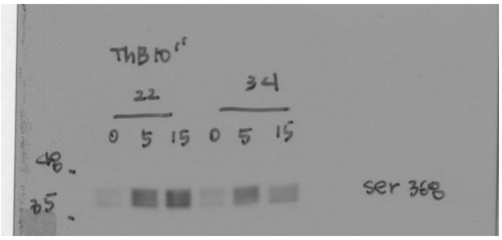
pCX43 (S368)

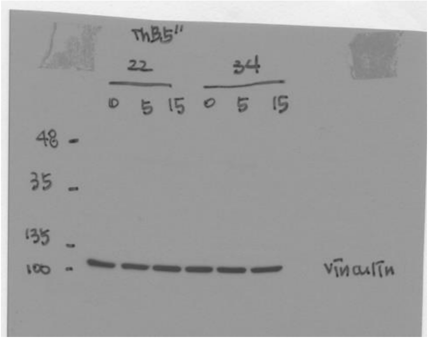
VINCULIN

Time post sync (hr) 30 42
ATP (min) 0 5 15 0 5 15

For ease of internal interpretation, we label "Time post sync (hr) 8 hr" as CT0. Therefore, the values 22 and 34 written on the scan file correspond to 30 and 42, respectively, in terms of "Time post sync (hr)."

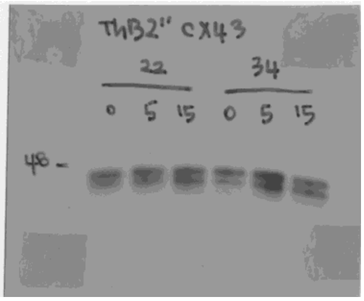
CX43

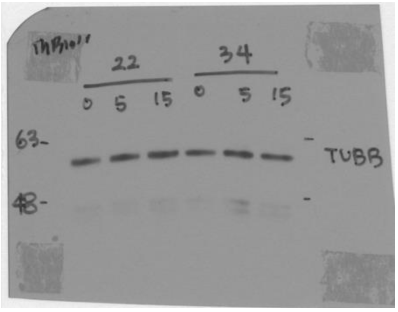
TUBB

Figure 6-source data 3 Original membranes corresponding to Figure 6, panel C, were used, with Gangnam-stained molecular weight markers.
