# Supplementary material for: Circadian regulation of endoplasmic reticulum calcium response in cultured mouse astrocytes
Source: eLife. 2024 Nov 27;13:RP96357. doi: 10.7554/eLife.96357 (PMC11602189; doi:10.7554/eLife.96357)
Supplement: Figure 6—source data 7. [file elife-96357-fig6-data7.zip › Figure 6-source data 7.pdf]

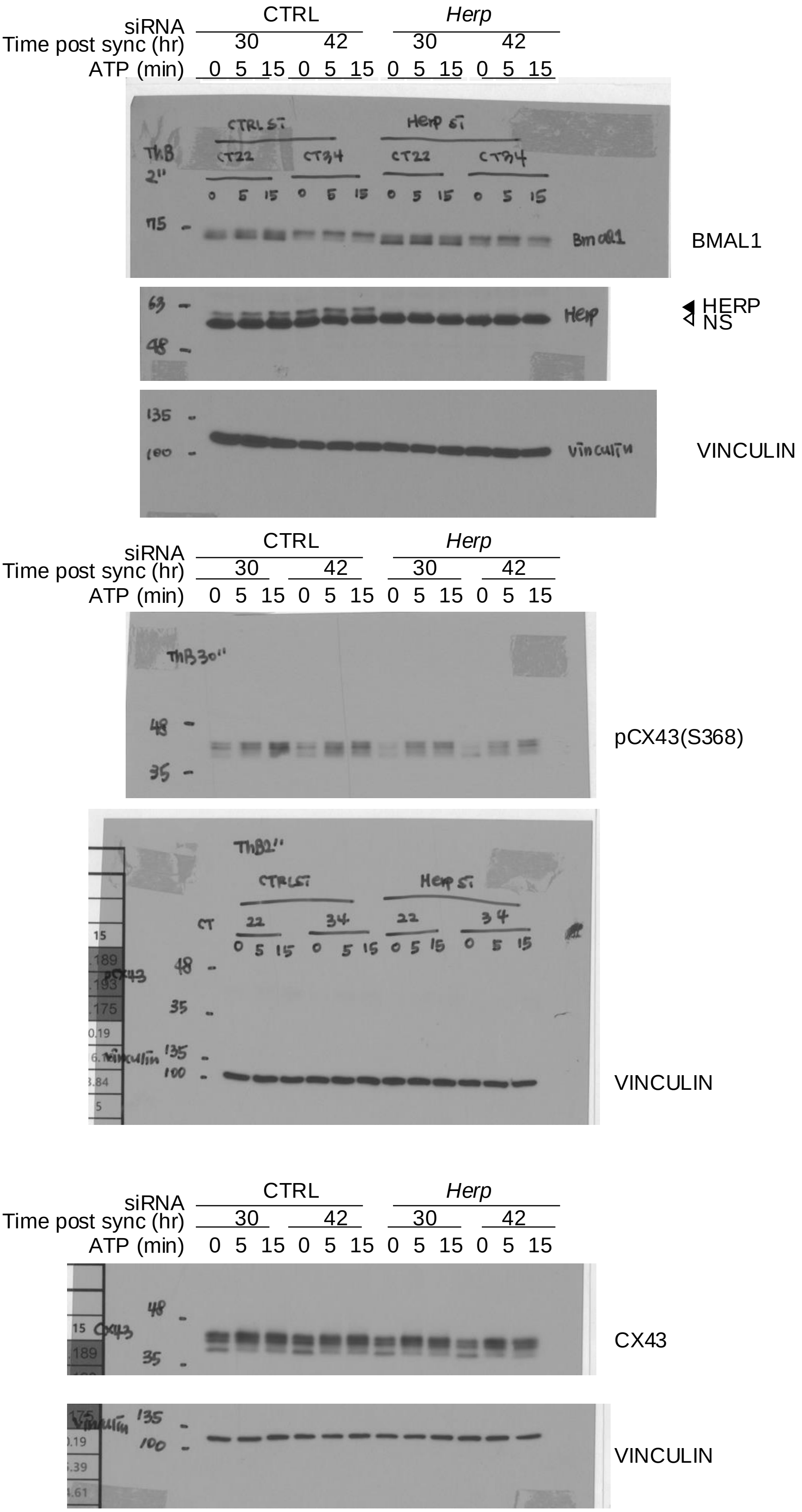

Figure 6-source data 7 Original membranes corresponding to Figure 6, panel G, were used, with Gangnam-stained molecular weight markers.
