# Supplementary material for: Circadian regulation of endoplasmic reticulum calcium response in cultured mouse astrocytes
Source: eLife. 2024 Nov 27;13:RP96357. doi: 10.7554/eLife.96357 (PMC11602189; doi:10.7554/eLife.96357)
Supplement: Supplementary file 2. — (A) A table of transcripts associated with the "Calcium ion homeostasis" GO term from the GO Biological Process analysis, ordered by ascending MetaCycle p-values. (B) A comprehensive list of all non-canonical E-boxes identified in the 6 kb upstream region of Herp through non-canonical E-box analysis. (C) Primer sequences used for quantitative RT-PCR. [file elife-96357-supp2.docx]

**Supplementary File 2**

**Supplementary File 2A. Calcium ion homeostasis**

|  | MetaCycle | | BioCycle | |  |
| --- | --- | --- | --- | --- | --- |
| Gene | p value | Amplitude | p value | Amplitude | Average TPM^a^ |
| *Herp* | 2.14E-09 | 11.66 | 0.00000001 | 11.35 | 71.13 |
| *Slc4a11* | 3.22E-09 | 1.48 | 0.00000100 | 1.50 | 6.68 |
| *Sord* | 3.60E-08 | 3.60 | 0.00002116 | 4.22 | 42.26 |
| *Kcnh1* | 5.50E-06 | 0.52 | 0.00000729 | 0.40 | 0.96 |
| *Anxa7* | 6.04E-06 | 4.44 | 0.00001444 | 4.23 | 102.07 |
| *Cx3cl1* | 3.13E-05 | 8.76 | 0.00019881 | 8.00 | 80.19 |
| *Pik3r1* | 5.21E-05 | 2.75 | 0.00126025 | 3.17 | 35.69 |
| *Hfe* | 0.000105052 | 2.94 | 0.00001600 | 2.95 | 32.10 |
| *Nr4a3* | 0.000106683 | 2.17 | 0.00064009 | 1.94 | 19.54 |
| *Spp1* | 0.000111664 | 31.47 | 0.00978121 | 41.58 | 168.53 |
| *Nppc* | 0.000162403 | 1.71 | 0.00017424 | 2.02 | 11.79 |
| *Sumo1* | 0.000462997 | 11.16 | 0.01687401 | 10.88 | 128.46 |
| *Tns2* | 0.000494944 | 1.01 | 0.00220900 | 1.21 | 13.15 |
| *Steap3* | 0.00050386 | 3.35 | 0.00494209 | 2.73 | 24.42 |
| *Capn1* | 0.000693268 | 2.57 | 0.00368449 | 2.49 | 48.74 |
| *Syt1* | 0.000707495 | 0.40 | 0.00015129 | 0.29 | 2.42 |
| *Ank* | 0.000906762 | 12.09 | 0.00077284 | 11.49 | 220.39 |
| *Casq2* | 0.001722008 | 0.67 | 0.00036864 | 0.64 | 5.78 |
| *Kcnj10* | 0.003437489 | 41.65 | 0.00088804 | 35.76 | 485.66 |
| *Adora1* | 0.005727835 | 6.56 | 0.00000009 | 7.08 | 63.08 |
| *P2ry1* | 0.006372537 | 1.77 | 0.00013225 | 1.99 | 19.40 |
| *Pcgf2* | 0.020637656 | 1.69 | 0.00090601 | 1.40 | 31.54 |
| *Dgat2* | 0.032505924 | 1.49 | 0.00055225 | 1.61 | 15.48 |

^a^Of all Time-merged values

**Supplementary File 2B. *Herp* upstream 6kb region non-canonical E-Box**

| E-box type | start position | end position | Upstream position | sequence | position |
| --- | --- | --- | --- | --- | --- |
| CANNTG | 94380510 | 94380515 | -5928 | CATCTG | chr8:94380510-94380515 |
| CANNTG | 94381181 | 94381186 | -5257 | CAAGTG | chr8:94381181-94381186 |
| CANNTG | 94381199 | 94381204 | -5239 | CAGCTG | chr8:94381199-94381204 |
| CANNTG | 94381442 | 94381447 | -4996 | CAATTG | chr8:94381442-94381447 |
| CANNTG | 94381536 | 94381541 | -4902 | CAAGTG | chr8:94381536-94381541 |
| CANNTG | 94381563 | 94381568 | -4875 | CAGATG | chr8:94381563-94381568 |
| CANNTG | 94381689 | 94381694 | -4749 | CAAATG | chr8:94381689-94381694 |
| CANNTG | 94381721 | 94381726 | -4717 | CATCTG | chr8:94381721-94381726 |
| CANNTG | 94382162 | 94382167 | -4276 | CAATTG | chr8:94382162-94382167 |
| CANNTG | 94382173 | 94382178 | -4265 | CACCTG | chr8:94382173-94382178 |
| CANNTG | 94382368 | 94382373 | -4070 | CAGGTG | chr8:94382368-94382373 |
| NC-CAGCTT | 94382386 | 94382391 | -4052 | CAGCTT | chr8:94382386-94382391 |
| CANNTG | 94382714 | 94382719 | -3724 | CACCTG | chr8:94382714-94382719 |
| CANNTG | 94382896 | 94382901 | -3542 | CAGCTG | chr8:94382896-94382901 |
| CANNTG | 94383317 | 94383322 | -3121 | CATATG | chr8:94383317-94383322 |
| CANNTG | 94383445 | 94383450 | -2993 | CAGATG | chr8:94383445-94383450 |
| CANNTG | 94384166 | 94384171 | -2272 | CAAGTG | chr8:94384166-94384171 |
| CANNTG | 94384284 | 94384289 | -2154 | CATTTG | chr8:94384284-94384289 |
| NC-CACGTT | 94384539 | 94384544 | -1899 | CACGTT | chr8:94384539-94384544 |
| CANNTG | 94384874 | 94384879 | -1564 | CAAATG | chr8:94384874-94384879 |
| CANNTG | 94384902 | 94384907 | -1536 | CACATG | chr8:94384902-94384907 |
| CANNTG | 94385063 | 94385068 | -1375 | CAGCTG | chr8:94385063-94385068 |
| CANNTG | 94385102 | 94385107 | -1336 | CAAATG | chr8:94385102-94385107 |
| CANNTG | 94385331 | 94385336 | -1107 | CAGGTG | chr8:94385331-94385336 |
| CANNTG | 94385353 | 94385358 | -1085 | CATGTG | chr8:94385353-94385358 |
| NC-CAGCTT | 94385511 | 94385516 | -927 | CAGCTT | chr8:94385511-94385516 |
| CANNTG | 94385809 | 94385814 | -629 | CAGATG | chr8:94385809-94385814 |
| CANNTG | 94385910 | 94385915 | -528 | CACTTG | chr8:94385910-94385915 |
| NC-CACGTT | 94386384 | 94386389 | -54 | CACGTT | chr8:94386384-94386389 |

**Supplementary File 2C: Table of primers used in quantitative RT-PCR**

| Primers | SOURCE |
| --- | --- |
| Bmal1 F: CAC CTA ATT CTC AGG GCA G | Marcrogen |
| Bmal1 R: CAC CTA ATT CTC AGG GCA G AGA CTC GGA GAC AAA GAG G | Marcrogen |
| Nr1d1(Rev-erb α) F: AAT GTT CTG CTG GCA TGT C | Marcrogen |
| Nr1d1(Rev-erb α) R: GAA GTC TTC CCA GAT CTC CT | Marcrogen |
| Hprt F: GATTAGCGATGATGAACCAGGT | Marcrogen |
| Hprt R: CCTCCCATCTCCTTCATGACA | Marcrogen |
| Herp F: GGT GGA GGA AGA TGA TGA GAT AAA | Marcrogen |
| Herp R: CTC AGC GAG GAG TAG AAG TAA AG | Marcrogen |
